# Comparative study of flexural strength test methods on CAD/CAM Y-TZP dental ceramics

**DOI:** 10.1093/rb/rbv020

**Published:** 2015-10-14

**Authors:** Yongxiang Xu, Jianmin Han, Hong Lin, Linan An

**Affiliations:** ^1^Department of Dental Materials, Peking University School and Hospital of Stomatology, Beijing 100081, China;; ^2^National Engineering Laboratory for Digital and Material Technology of Stomatology, Peking University School and Hospital of Stomatology, Beijing 100081, China;; ^3^Department of Materials Science and Engineering, Advanced Materials Processing and Analysis Center, University of Central Florida, Orlando, FL 32816, USA

**Keywords:** CAD/CAM Y-TZP dental ceramics, flexural strength, test methods

## Abstract

Clinically, fractures are the main cause of computer-aided design and computer-aided manufacturing (CAD/CAM) 3 mol%-yttria-stabilized tetragonal zirconia polycrystal (Y-TZP) all-ceramic dental restorations failure because of repetitive occlusal loading. The goal of this work is to study the effect of test methods and specimen’s size on the flexural strength of five ceramic products. Both bi-axial flexure test (BI) and uni-axial flexure tests (UNI), including three-point flexure test (3PF) and four-point flexure test (4PF), are used in this study. For all five products, the flexural strength is as follows: BI > 3PF > 4PF. Furthermore, specimens with smaller size (3PF-s) have higher values than the bigger ones (3PF). The difference between BI and UNI resulted from the edge flaws in ceramic specimens. The relationship between different UNI (including 3PF-s, 3PF and 4PF) can be explained according to Weibull statistical fracture theory. BI is recommended to evaluate the flexural strength of CAD/CAM Y-TZP dental ceramics.

## Introduction

Computer-aided design and computer-aided manufacturing (CAD/CAM) dental ceramics are widely used in modern dentistry based on their excellent machinability, aesthetics, chemical stability and biocompatibility [1–6]. CAD/CAM dental ceramic, mainly 3 mol%-yttria-stabilized tetragonal zirconia polycrystal (Y-TZP) ceramics, has different compositions and fabrication techniques from traditional dental ceramic such as porcelain for veneering, injectable ceramics and castable ceramics [7–9]. Typically, CAD/CAM ceramic blank is fabricated by Y-TZP powder and a binder. The binder is later eliminated during the pre-sintering step. After that, a pre-sintered ceramic blank is milled to a restoration or prosthesis by CAD/CAM process. Finally, the restoration or prosthesis is sintered at high temperature with approximately 20% shrinkage [10]. Moreover, the translucency of Y-TZP ceramic is a major drawback for dental restorations or prosthesis. High translucency dental ceramic have been developed by additive components and heat treatments in the recent years [11, 12].

Clinical data on survival rates reveals that all-ceramic dental restorations are susceptible to fracture from repetitive occlusal loading [12, 13]. Therefore, *in vitro* characterization of dental ceramics must include a test method capable of accessing their fracture properties [14]. Flexural strength is generally considered a meaningful and reliable characterization parameter to assess the ceramics as they are much weaker in tension than compression [15, 16].

Two main techniques have been described to determine the flexural strength of ceramics: uni-axial flexure test (UNI) and bi-axial flexure test (BI) [17]. In UNI, beam-shaped specimens with a rectangular cross section is supported by two points and the load is applied vertically at either one point (three-point flexure test, 3PF) or two points (four-point flexure test, 4PF), respectively [10]. As an alternative to UNI, BI has been developed to measure the flexural strength of ceramics [18, 19]. BI includes piston-on-three-ball, piston-on-ring, ball-on-ring and ring-on-ring test methods. In such tests, a thin disc is supported by a ring (or three balls) near its periphery and loaded through a smaller coaxial ring, a piston or a ball in its central region [20, 21]. Many researchers have studied the relationship between 3PF, 4PF and BI for the dental composites, cements, veneered ceramic and so on [22, 23].

Little research has been done to compare and analyze the difference and correlation between these test methods for CAD/CAM Y-TZP dental ceramics although both UNI (3PF and 4PF) and BI (piston-on-three-ball) have been recommended to evaluate dentistry-ceramic materials in International Organization for Standardization (ISO) 6782 [24]. Different flexural strength values have been given by different researchers and manufactures for the same product, just because of the difference of test methods and specimens used. All of these make it confused for dentist to choose and apply the suitable product in dental clinic.

In light of the problems described above, the goal of this study is to compare and analyze the relationship between these test methods applied to CAD/CAM Y-TZP dental ceramics using one-way analysis of variance (ANOVA), Weibull statistics and fractographic analyses. Five commercial ceramic products from three manufactures are used, including two mainly types: traditional ceramics and high translucency ceramics.

## Materials and methods

### Materials

Five CAD/CAM dental ceramic products were studied in this research, as listed in Table 1. Among these, three samples (UH, LF and KE) were traditional Y-TZP ceramics and two samples (UT and LP) were high translucency Y-TZP ceramics. Specimens were prepared according to the instruction of the manufacturers and further processed according to ISO 6872.

**Table 1.** CAD/CAM Y-TZP dental ceramics used in this study

Abbreviation	Materials	Lot No.	Manufacture
UH	UPCERA HT	L2121121001	Shenzhen Upcera Co., Ltd, Guangdong, China
UT	UPCERA ST	L2121121013	Shenzhen Upcera Co., Ltd, Guangdong, China
LP	LAVA Plus	468553	3M ESPE, St. Paul, MN
LF	LAVA Frame	467091	3M ESPE, St. Paul, MN
KE	Kavo Everest	101545349	KaVo Dental GmbH, Biberach, Germany

### Methods

#### 3PF & 3PF-s

The sample holder included two support rollers and one loading roller, and the diameter of roller was 5 mm. Load was applied at the midpoint of the specimens. 30 beam-shaped specimens with a final size 4.0 × 3.0 × 42 mm (3PF) and 4.0 × 1.2 × 14 mm (3PF-s) were produced, separately. The corresponding sample holders with a span between the two support rollers of 40 mm and 12 mm were used, respectively.

#### 4PF

The sample holder was 4-point-1/4-point fixture, including two support rollers (40 mm between their centers) and two loading rollers (20 mm between their centers) and the diameter of rollers was 5 mm. Thirty beam-shaped specimens with the same final size as 3PF were prepared.

#### BI

The sample holder was a piston-on-three-ball test fixture. The balls had a diameter of 3.2 mm and were arranged in an angle of 120° to each other on a circle of 10 mm in diameter. The load was applied with a flat punch with a diameter of 1.4 mm at the center of the specimen. Thirty disc specimens with the size ø12 × 1.2 mm were prepared.

#### Testing process

All the specimens were tested at a cross-head speed of 0.5 mm/min in a universal testing machine (Model 5565, Instron Corp., Canton, MA) and the flexural strength was calculated as described in ISO 6872: 2008.

### Statistical analysis

Data obtained from the flexural tests were analyzed with one-way ANOVA (SPSS Inc., Chicago, IL) for differences between five ceramics in each method and further between different methods with the same product (*P* = 0.05).

Weibull analysis was further adopted as a complement due to its capability to analyze material’s phenomena or properties represented by a symmetrical and asymmetrical data set.

### Fractographic analysis

The morphology of fractured specimens was observed and the representative specimens were selected for scanning electron microscope (SEM) analysis (EVO 18, Carl Zeiss, Oberkochen, Germany).

## Results

### Flexural strength of ceramics

**Figure 1.**
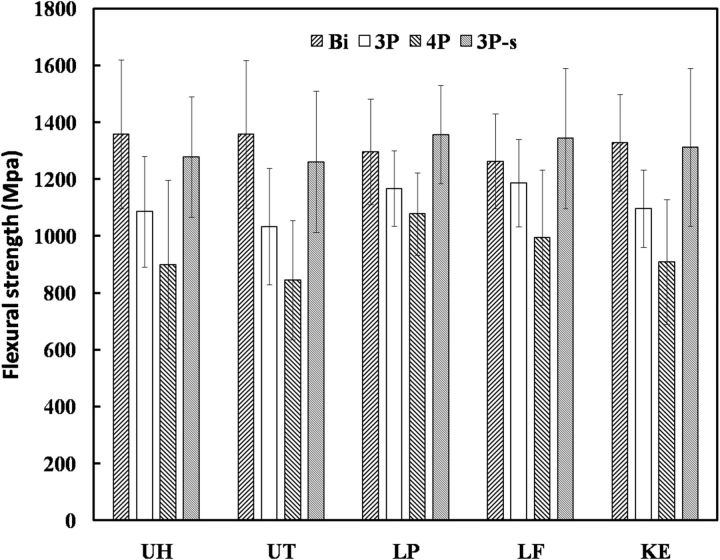
Mean values and standard deviations of the flexural strength of five ceramics (UH, UT, LP, LE and KE) tested by different methods: BI, 3PF, 4PF and three-point flexure test of specimens with smaller size (3PF-s).

Mean values and standard deviations of all groups were shown in Fig. 1. The flexural strength from different test methods were as follows: BI > 3PF > 4PF. Furthermore, the flexural strength of specimens with smaller size (3PF-s) was higher than the bigger ones (3PF).

**Figure 2.**
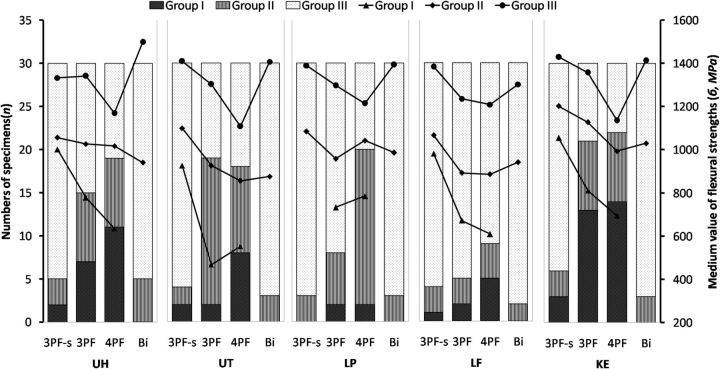
Numbers of specimens and medium values of flexural strengths for different fractured fragments after test of five ceramics (UH, UT, LP, LE and KE) tested by 3PF-s, 3PF 4PF and BI: Group I with two fractured fragments, Group II with three fractured fragments and Group III with more than three fractured fragments.

According to the quantity of fractured fragments after testing, the specimens were divided into three groups: Group I with two fractured fragments, Group II with three fractured fragments and Group III with more than three fractured fragments. The UNI specimens included all the three groups and the flexural strength from low to high was Group I, Group II and Group III. Nevertheless, there are only two groups (Group II and Group III) for BI specimens, and the flexural strength of Group II was also lower than Group III (Fig. 2). For all the samples, the higher the quantity of fractured fragments, the higher the flexural strength.

One-way ANOVA indicated that there was significant difference between ceramics using different test methods, as listed in Table 2. In detail, there was significant difference for three pairs of ceramics using 3PF and 4PF. In addition, there was significant difference for two pairs using 3PF-s. No significant difference between all five ceramics was detected by BI. For all five ceramics, there was significant difference between different specimen size (3PF and 3PF-s) (*P* < 0.005). The mean values of coefficient of variation (C.V.) for five ceramics were 3PF-s (12.5%), BI (15.8%), 3PF (21.4%) and 4PF (23.6%), separately.

**Table 2.** one-way ANOVA statistics analysis for five ceramics

	UNI			BI
	3PF-s	3PF	4PF	
*F*	2.415	3.584	6.979	1.12
*P*	0.051	0.008	0	0.349
*	UT&LP	UT&LF	UH&LP	No
	UT&KE	UT&LP	UT&LP	
		LP&KE	LP&KE	

*Significant difference between two dental ceramics.

**Figure 3.**
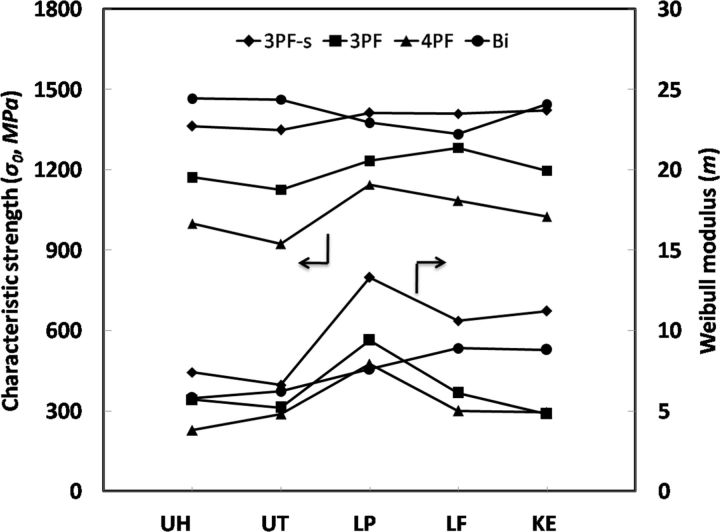
Characteristic strength (*σ*_0_, MPa) and Weibull modulus (*m*) of five ceramics (UH, UT, LP, LE and KE) tested by 3PF-s, 3PF 4PF and BI.

The characteristic strength (*σ*_0_) and Weibull modulus (*m*) parameters obtained by the five ceramics in the UNI and BI were shown in Fig. 3. For all five ceramics, the *σ*_0_ from high to low was 3PF-s, 3PF and 4PF, the same as mean values in Fig. 1. In the meantime, the mean values of Weibull modulus (*m*) from high to low were also 3PF-s, BI, 3PF and 4PF (Table 2).

### Fractographic analysis

**Figure 4.**
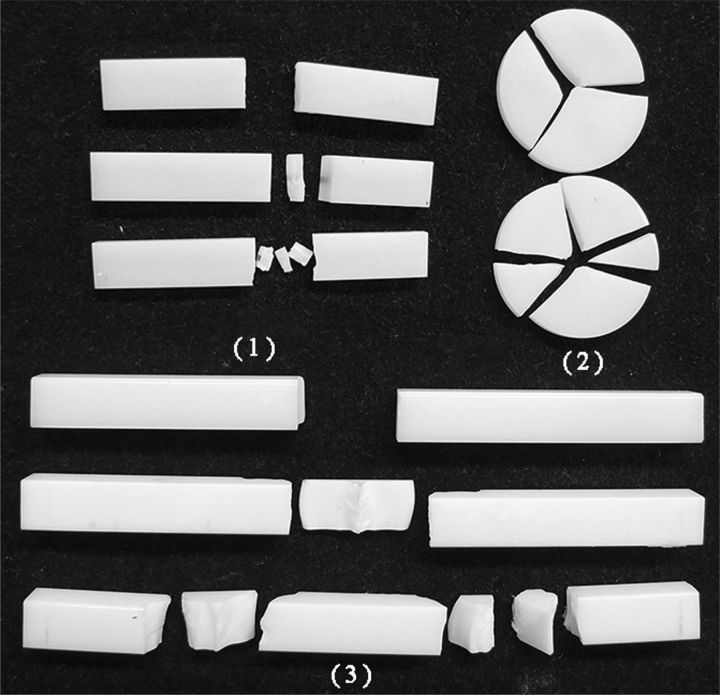
The examples of fracture morphology of ceramics tested by (1) 3PF-s, (2) BI and (3) 4PF. There was Group I, Group II and Group III from top to bottom for each method.

**Figure 5.**
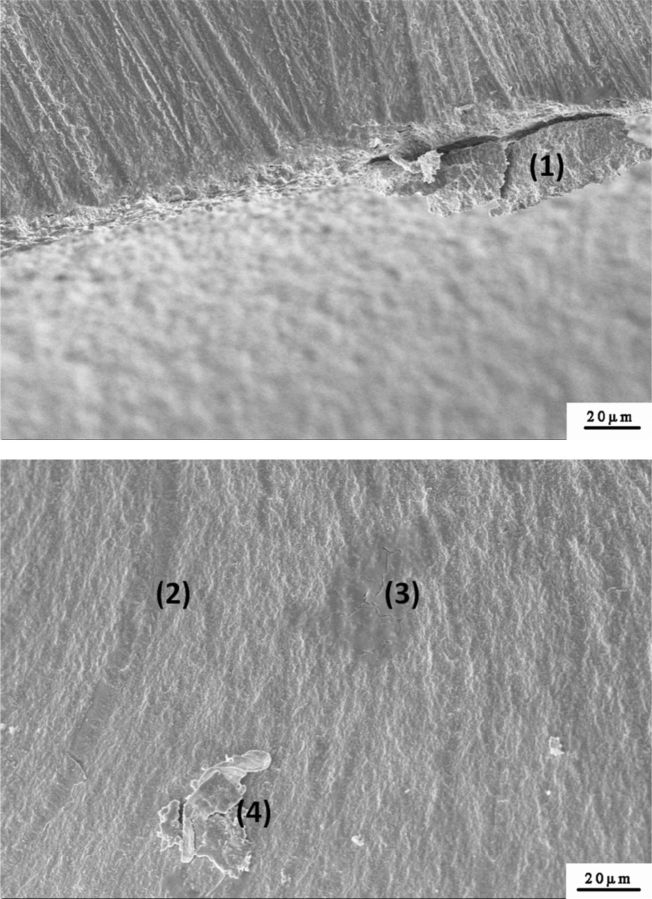
Scanning electron micrographs of the fractured surfaces of the CAD/CAM Y-TZP dental ceramics: (1) surface flaw, (2) hackle line, (3) interior pore and (4) interior flaw.

For all specimens after testing, fractured fragments fit perfectly with no signs of macroscopic plastic deformation in the test process. Obvious flaws were observed on the surface of the specimens by stereomicroscope analysis. As to the specimens with three fractured fragments, a triangle fractured fragments was found in the middle of specimens (Fig. 4). The stereomicroscope analysis also revealed that fracture origin was located on the surface opposed to loading. Fractographic features, such as hackle lines, were easily found on the fractured surfaces in SEM. The flaws and pores were observed inside the ceramics by SEM at greater magnifications (Fig. 5).

## Discussion

According to ‘weakest link theory’, flexural strength is strongly influenced by variations in flaw population of the specimens and fracture happens as soon as the weakest of these flaws starts to grow [25]. In this study, the less of the quantity of fractured fragments indicate the weaker of the flaws than the higher ones, which further result to the lower flexural strength. Furthermore, because of the bigger effective volume of material subjected to the same bending moment in 4PF, there is a higher probability that defects exists in this volume than 3PF. Consequently, 4PF gives a lower strength value than the 3PF, as showed in finite element analysis [10]. For the same test method, specimens with smaller size (3PF-s) show a higher strength values and less variation than the bigger ones (3PF). The lower effective volume of 3PF-s than 3PF can be used to explain these results. A quantitative relationship between the strength value of 3PF-s, 3PF and 4PF can be established by Weibull statistics [23]. Beam-shaped specimens often have edge flaws, which act as stress concentration sites and lead to undesirable edge failures, instead of having a fracture that originates from a material’s intrinsic flaw [26]. As a result, uni-axial beam-shaped specimens resulted to a lower strength values than the actual strength of the materials [27].

BI has several advantages over UNI because multi-axial stress states are produced and edge failures are eliminated [28, 29]. In BI, a thin disc specimen is subjected to a bi-axial moment in its central region and the stresses are bi-axial in this region [21]. The maximum tensile stress occurs at the center of the surface opposite to load application and, consequently, edge flaws do not influence the results [30]. Studies comparing UNI and BI in composites [22, 31], glass ionomer cements [32] and ceramics [22, 33–35] show higher strength values for BI than UNI, in accordance with this study. Both coefficient of variation (C.V) and Weibull modulus (*m*) show less variation in data of BI than UNI. It is very difficult to prepare specimens for pure tensile tests because Y-TZP dental ceramics is a brittle material. Processing flaws, which mostly concentrated upon the surface of specimens, facilitate crack initiation and fracture at end [22]. Comparing with UNI specimens, BI specimens were not influenced by the presence of edge flaws since the disc edges are located in a low stress area [36]. As a consequence, BI has high value and less dispersion than UNI. Furthermore, the effect of effective volume was another factor that should be consider in the further works.

Both 3PF and 4PF showed that there is significant difference between three pairs’ ceramics. However, no significant difference is detected for BI. As discussed above, the BI is more reliable than UNI [37]. Therefore, the significant difference detected in 3PF and 4PF maybe from the surface flaws in manufacturing process and not from intrinsic strength of ceramics.

The analysis of the fractured morphology allow for the observation of fractographic features commonly found in ceramic specimens [21]. The longitudinal stress in the beam-shaped specimens of UNI is tensile at their lower surfaces and compressive at their upper surfaces. The ceramics fracture begins from their lower surfaces and then the upper surfaces as they are much weaker in tension than compression [15, 16]. Consequently, a triangle fracture fragment in the middle of specimens is formed in test process.

Flaws and crack are identified in the SEM images and also described in a previous study [38]. Fractographic analysis of the flexure test specimens determines that the predominant flaw type is volume distributed porosity or agglomerates associated with porosity [39]. The flaws and pores inside the ceramics can explain that the testing values were 0.1% to 10% of theoretical values by Griffith–Orowan fracture theory [40].

Furthermore, researches show that the bi-axial stress state is better suited to conservative strength design, with practical similarities to stresses which occur in thin tooth section [41].

## Conclusions

Within the limitations of this study, the following conclusions were drawn:

1. As the specimen’s sizes and preparation process recommended in ISO 6872, the flexural strength values from different test methods were as follows: BI > 3PF > 4PF.
2. The relationship between different uni-axial test methods (3PF & 4PF) was analyzed by Weibull statistical fracture theory, the same as relationship between different specimen sizes (3PF & 3PF-s).
3. The difference between UNI and BI mainly resulted from the effect of edge flaws and BI was a more reliable method than UNI.
4. Both flaws and pores were observed on the surface and interior of these dental ceramics, which resulted to a lower flexural strength than theoretical value.
